# The impact of diagnostic microbiology on de-escalation of antimicrobial therapy in hospitalised adults

**DOI:** 10.1186/s12879-020-4823-4

**Published:** 2020-02-03

**Authors:** William L. Hamilton, Sacha-Marie Pires, Samantha Lippett, Vikesh Gudka, Elizabeth L. A. Cross, Martin J. Llewelyn

**Affiliations:** 1 Brighton and Sussex University Hospitals NHS Trust, Brighton, UK; 2 Cambridge University Hospitals NHS Foundation Trust, Cambridge, UK; 3 Brighton and Sussex Medical School, University of Sussex, Brighton, UK

**Keywords:** Antimicrobial resistance, Diagnostic microbiology, Antibiotics, Prescribing, Antimicrobial stewardship

## Abstract

**Background:**

Minimising antimicrobial overuse is needed to limit antimicrobial resistance. There is little evidence on how often microbiological testing informs antimicrobial de-escalation (e.g. stopping, shortening duration, switching to narrower spectrum or intravenous to oral switch) at 48–72 h “review and revise”. We performed a patient level analysis of diagnostic microbiology and antimicrobial prescribing to determine the impact of microbiology results on antimicrobial review outcomes.

**Methods:**

Antimicrobial prescribing data were collected for hospitalised adults from across Brighton and Sussex University Hospitals NHS Trust using routine monthly audits of prescribing practice from July 2016 to April 2017. Microbiology testing data for cultures of blood, urine, sputum and cerebrospinal fluid (CSF) were gathered from the hospital pathology database and linked to prescriptions with matching patient identification codes. Antimicrobial prescriptions were grouped into “prescription episodes” (PEs), defined as one or more antimicrobials prescribed to the same patient for the same indication. Medical records were reviewed for all PEs with positive microbiology and a randomised sample of those with negative results to assess the impact of the microbiology result on the antimicrobial prescription(s).

**Results:**

After excluding topical and prophylactic prescriptions, data were available for 382 inpatient antimicrobial prescriptions grouped into 276 prescription episodes. 162/276 (59%) had contemporaneous microbiology sent. After filtering likely contaminants, 33/276 (12%) returned relevant positive results, of which 20/33 (61%) had antimicrobials changed from empiric therapy as a result with 6/33 (18%) prompting de-escalation. Positive blood and CSF tended to have greater impact than urine or sputum cultures. 124/276 (45%) PEs returned only negative microbiology, and this was documented in the medical notes less often (9/40, 23%) than positive results (28/33, 85%). Out of 40 reviewed PEs with negative microbiology, we identified just one (~ 3%) in which antimicrobials were unambiguously de-escalated following the negative result.

**Conclusions:**

The majority of diagnostic microbiology tests sent to inform clinical management yielded negative results. However, negative microbiology contributed little to clinical decision making about antimicrobial de-escalation, perhaps reflecting a lack of trust in negative results by treating clinicians. Improving the negative predictive value of currently available diagnostic microbiology could help hospital prescribers in de-escalating antimicrobial therapy.

## Introduction

Reducing antimicrobial over prescribing is key to mitigating the threat of antimicrobial resistance [1, 2]. While hospital use accounts for < 20% of prescribed antimicrobials in most advanced healthcare systems, around two-thirds of prescriptions for ‘broad-spectrum’ agents are made in this setting [3]. Hospital clinicians prioritise anticipated benefits of starting empiric antimicrobial therapy when infection may be present over risks of antimicrobial exposure. Controlling overuse depends on regular “review and revise” of patients on antimicrobials with a view to de-escalation (i.e. switching to “narrower spectrum” agents and/or reducing the number of agents prescribed, switching from intravenous to oral route, shortening treatment duration or stopping antimicrobial therapy). Strategies such as “Start Smart then Focus” in the UK [4] and “Antibiotic Timeouts” in the USA [5] utilise diagnostic information to support de-escalation decisions made at around 48–72 h. Provision of timely and reliable patient level diagnostic results to support “review and revise” is one of the key ways in which microbiology laboratories can contribute to antimicrobial stewardship [6]. However, while reductions in UK National Health Service (NHS) antibiotic prescribing have been achieved in primary care, hospital prescribing increased by 7.7% in England between 2013 and 2017 [3].

The extent to which diagnostic microbiology informs antimicrobial decision making in individual patients is limited by pre-analytic factors (most notably whether samples are taken), analytic factors (time, test performance) and post-analytic factors (reporting, interpretation). There is a lack of evidence for which of these really matters to inform strategies to maximise the impact of diagnostic testing. We performed a patient level analysis of diagnostic testing and prescribing practice in our hospital to determine what impact diagnostic results have on antimicrobial de-escalation decision making.

## Methods

### Antimicrobial prescriptions

**Fig. 1:**
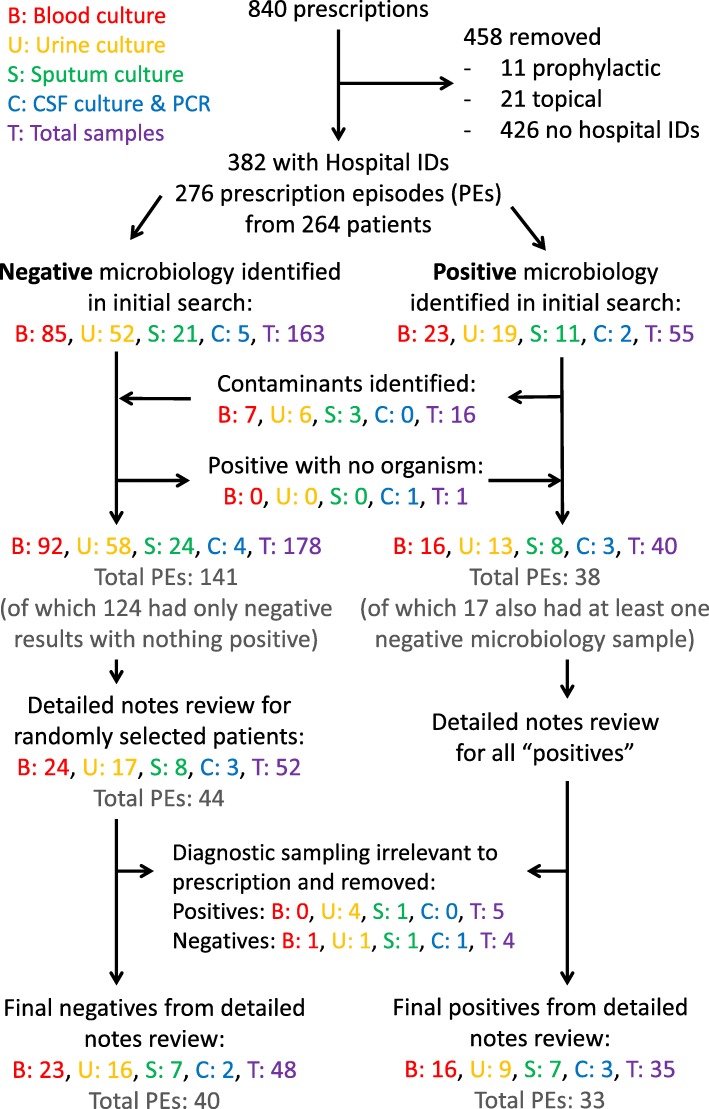
Flow chart of antimicrobial prescription filtering and linking to diagnostic microbiology. Of 840 prescriptions collected by pharmacists, samples were excluded if they were prophylactic (*n* = 11; breakdown: 7 peri-operative or otherwise surgical and 4 medical), topical (*n* = 21), or lacking unique patient hospital identification numbers, IDs (*n* = 426). The 382 remaining prescriptions were grouped into 276 “Prescription Episodes” (PEs) of antimicrobials given for the same patient illness (see main text). Contemporaneous diagnostic microbiology from blood, urine, sputum or CSF were linked to prescriptions with matching hospital IDs. Contaminants (as initially flagged up in the microbiologist comment on the result, then confirmed on case review by authors WLH and SP) were transferred from “positive” to “negative” and a single CSF sample was moved from “negative” to “positive” due to elevated white cell count without any microorganism identified. All PEs with positive pathogenic microbiology (*n* = 38) and a randomised sample of the 124 PEs with negative microbiology (*n* = 44) were reviewed, with 9 PEs removed at this stage due to the linked microbiology sample being deemed irrelevant to the antimicrobial prescription. This left a final set for detailed case review of 33 PEs with positive microbiology and 40 with negative microbiology

Antimicrobial prescribing data for adult inpatients were extracted from routine pharmacy audits of prescribing quality conducted across the Brighton and Sussex University Hospitals NHS Trust (BSUH), from July 2016 to April 2017, excluding September 2016 when staff were unavailable. Sampling included acute, medical, surgical and intensive care wards. Prescriptions were removed if they were for topical agents, prophylactic use or if no unique patient hospital identification (ID) was recorded (Fig. 1). Antimicrobial indications were grouped into major categories: respiratory, urinary, sepsis unclear source, skin and soft tissue, abdominal, other, and not documented.

We grouped prescriptions into “Prescription Episodes” (PEs), defined as one or more antimicrobials prescribed for the same patient during the same time period for the same illness (where the latter could be determined). For example, amoxicillin and doxycycline treating pneumonia would constitute a single PE; whereas a patient given flucloxacillin, teicoplanin and clindamycin for osteomyelitis and trimethoprim for an intercurrent urinary tract infection (UTI) would count as two PEs (one for osteomyelitis, the other for UTI). Where no indication was documented, prescriptions from the same patient were assumed to be from separate admissions (and so count as distinct PEs) if they were prescribed ≥ month apart.

### Microbiology results

For each patient ID, microbiology results for blood, urine, sputum or cerebrospinal fluid (CSF) samples sent within +/− 7 days of the prescription date were pulled from the hospital pathology system ‘Winpath’. Where prescription dates were unrecorded, test results were analysed for prescriptions from the same month or the 3 days at the start and end of the adjacent months. Microbiology results were considered ‘positive’ if clinically significant growth of a recognised pathogen was recorded. Otherwise, culture negative samples and cultures yielding organisms likely to be contaminants were considered ‘negative’ (Fig. 1). Contaminants were initially flagged up in the microbiologist comments section on the result (also viewable by treating clinicians), and confirmed on case review by authors SP and WLH.

A single CSF sample with no microorganism identified on Gram stain or CSF culture was considered ‘positive’ due to significantly elevated CSF white cell count in an intubated patient with external ventricular drain in situ and new onset fever. We note that diagnosis and management of healthcare-associated ventriculitis is complex [7]; as our study focused on decision making by antimicrobial prescribers, we interpreted this CSF result in the same way as the treating clinicians had done i.e. a positive result suggesting bacterial ventriculitis.

Regarding microbiology testing methods at BSUH during the study: the laboratory routinely used MALDI-TOF identification for cultures that flagged positive following initial Gram stain. We did not assess the impact of active surveillance cultures (e.g. swabs screening for MRSA colonisation), and none of the antimicrobial prescriptions in our dataset were for MRSA decolonisation treatment.

### Impact of diagnostic testing on antimicrobial prescription review

We reviewed case records for all patients with positive diagnostic results and a random sample of ~ 25% patients with negative results (randomisation was performed in the R programming language). Reviews were conducted by authors WLH and SP using paper medical notes, electronic discharge summaries and electronic test results (blood tests, microbiology *etc*), with ambiguous cases resolved through group discussion. Records were excluded from further analysis if the microbiology result was deemed irrelevant to the indication for antimicrobial treatment (for example, doxycycline prescribed to treat community acquired pneumonia, with a microbiology urine sample sent for query UTI).

Data were collected on microbiology result documentation in the medical records, involvement of infectious disease and/or microbiology teams, antimicrobial prescription review decisions, and the likelihood that the microbiology result influenced prescribing decisions (see Additional file 1). We defined antimicrobial “escalation” as adding agent(s), changing to “broader” cover, switching from oral to intravenous, or lengthening treatment duration; “de-escalation” was defined as the inverse of these.

### Statistics

All data manipulations and analyses were performed in the programming language R and using the *dplyr* package. For important findings we quote exact binomial 95% confidence intervals (95%CI) and *P*-values, calculated using the R package *binom*.

## Results

### Antimicrobial prescriptions

After filtering (Fig. 1), 382 antimicrobial prescriptions were grouped into 276 Prescription Episodes (PEs) from 264 patients. The vast majority of antimicrobial prescriptions were antibacterials (376/382, 98%), with the remainder being antifungals (5/382, 1.3%) and antiviral (1/382, 0.3%). There were no antiretrovirals or anti-TB medications included. The majority of prescriptions were intravenous (245/382, 64%) and from general medical wards (230/382, 60%) (Additional file 2: Tables S1–S4). The most commonly prescribed antimicrobials were co-amoxiclav (68/382, 18%), piperacillin-tazobactam (58/382, 15%) and amoxicillin (40/382, 11%). The most commonly documented indications were respiratory (134/382, 35%), urinary (51/382, 13%) and sepsis unclear source (51/382, 13%). 34/382 (8.9%) PEs were for “other” indications, broken down into: osteomyelitis (*n* = 14), central nervous system (CNS) infections (*n* = 7), endocarditis (*n* = 6), ear nose & throat (ENT, *n* = 2), oesophageal candidiasis (*n* = 2), oral infections (*n* = 2) and septic arthritis (*n* = 1).

Antimicrobial indication and duration were documented in 92 and 80% of prescriptions for which audit data were recorded, and complied with Trust guidelines in 82 and 81%, respectively (after removing prescriptions with incomplete pharmacy data collection, Additional file 2: Table S5). Data on 72-h reviews were recorded for 290/382 (76%) prescriptions, of which the main outcomes were continue (141/290, 37%), stop (76/290, 20%) and intravenous to oral switch (47/290, 12%) (Additional file 2: Table S6).

### Diagnostic microbiology and impact on prescription review

162/276 (59, 95%CI 53–65%) PEs had contemporaneous microbiology results identified from the hospital pathology database. Where a prescription date was recorded, there was a median of 2 days (interquartile range 1–4) between the prescription and the microbiology sample being taken. “Sepsis unclear source” was the most likely indication to have microbiology sent (27/35, 77%), while “skin and soft tissue” was least (14/34, 41%) (Table 1). 124/276 (45, 95%CI 39–51%) PEs had exclusively negative microbiology results; 21 had exclusively positive results and 17 had both positive and negative results, which were analysed together as “positives”.

**Table 1:** Impact of diagnostic microbiology on antimicrobial prescribing for different prescription episode indications

Indication type	Total prescriptions analysed	Prescription episodes (PE)	PE with micro: all*	PE with relevant positive micro *	Positive micro result documented **	Positive micro with ID/micro team involved **	Positive micro where Abx were affected**	Positive micro where Abx were de-escalated**	PE with only negative micro*	Negative micro reviewed & relevant	Reviewed negative micro result documented***	Reviewed negative micro result affected Abx
Respiratory	134	90	56 (62%)	10 (11%)	7 (70%)	7 (70%)	5 (50%)	1 (10%)	45 (50%)	19	4 (21%)	0
Urinary	51	44	28 (64%)	8 (18%)	7 (88%)	4 (50%)	3 (38%)	2 (25%)	19 (43%)	4	1 (25%)	0
Sepsis unclear source	51	35	27 (77%)	4 (11%)	4 (100%)	4 (100%)	4 (100%)	2 (50%)	22 (63%)	4	1 (25%)	1
Skin and soft tissue	44	34	14 (41%)	1 (3%)	1 (100%)	1 (100%)	1 (100%)	0	13 (38%)	3	1 (33%)	0
Abdominal	27	18	11 (61%)	2 (11%)	2 (100%)	2 (100%)	2 (100%)	0	8 (44%)	3	1 (33%)	0
Other	34	22	11 (50%)	5 (23%)	5 (100%)	5 (100%)	5 (100%)	1 (20%)	5 (23%)	4	1 (25%)	0
No indication documented	41	33	15 (45%)	3 (9%)	1 (33%)	0	0	0	12 (36%)	3	0	0
TOTAL	382	276	162 (59%)	33 (12%)	27 (82%)	23 (70%)	20 (61%)	6 (18%)	124 (45%)	40	9 (23%)	1 (2.5%)

Table shows the total prescriptions sampled for different indications, the Prescription Episodes analysed (PEs, defined in main text), and various properties of PEs with positive and negative diagnostic microbiology found on detailed notes review. For PEs with positive microbiology, data were collected on whether the microbiology result was documented in the medical records, whether infectious diseases and/or microbiology teams were involved (either by telephone or patient review), whether the antimicrobial regimen was changed as a result of the microbiology and whether that change constituted escalation or de-escalation. For a random sample of PEs with negative microbiology, data were collected on whether the result was acknowledged in the medical records and whether this caused antimicrobial de-escalation. *ID* Infectious Diseases team. * = Denominator for percentages is all PEs for that indication; ** = Denominator for percentages is all PEs with pathogenic positive microbiology; *** = Denominator for percentages is reviewed PEs with negative microbiology

After removing positives with irrelevant microbiology, 33/276 (12, 95%CI 8.4–16%) of all PEs had a pathogenic microbiological diagnosis available to guide prescribing (Table 1), or 33/162 (20, 95%CI 14–27%) of PEs for which any samples had been sent for testing. “Skin and soft tissue” was the least likely indication to return relevant positive microbiology (1/34, 3%), while “other” was most likely (5/22, 21% - mainly driven by endocarditis, osteomyelitis and CNS infections). Review of the medical records for all 33 positives suggested that the microbiology result affected the antimicrobial decision making in 20 PEs (7% of total, 61% of PEs with relevant positive microbiology), but in only 6 PEs (2% of total, 18% of positives) did the positive microbiology lead to antimicrobials being “de-escalated” (Additional file 2: Tables S7–S8). Blood cultures and CSF, if positive, were more likely to affect the antimicrobial regimen (15/16, 94% and 3/3, 100%, respectively) than urine or sputum (1/9, 11% and 2/7, 29%, respectively) (Table 2). Positive blood cultures had other impacts on clinical management, such as prompting echocardiography to exclude endocarditis.

**Table 2:** Impact of positive microbiology on antimicrobial prescribing for different microbiological sampling types

Microbiology sample type	PE with relevant positive micro	Positive micro result documented*	Positive micro with ID/micro team involved*	Positive micro where Abx were affected*	Positive micro where Abx were de-escalated*
Blood	16	16 (100%)	16 (100%)	15 (94%)	6 (38%)
Urine	9	5 (56%)	2 (22%)	1 (11%)	1 (11%)
Sputum	7	5 (71%)	4 (57%)	2 (29%)	0
CSF	3	3 (100%)	3 (100%)	3 (100%)	0
Total	35	29 (83%)	25 (71%)	21 (60%)	7 (20%)

Table shows the Prescription Episodes (PEs, defined in main text) with positive diagnostic microbiology broken down by microbiological sampling type (blood, urine, sputum, cerebrospinal fluid (CSF)). Compared with urine or sputum, positive blood and CSF cultures were more likely to be documented in the medical records, to have infectious diseases and/or microbiology teams involved, and to result in the antimicrobial regimen being altered (*P*<10^-4^ for pairwise comparisons between blood cultures and urine or sputum cultures, exact binomial test after Bonferroni correction for multiple comparisons). *ID* Infectious Diseases team, *ABx* Antimicrobials; * = Denominator for all cases is the “PE with relevant positive micro” column. Note that 33 PEs had significant positive microbiology; the total shown of 35 is because two patients had two positive culture types (blood and urine or blood and sputum) and these were counted separately for this table

Negative microbiology results were more common (141/276, 51% (95% CI 45–57%) of PEs returning at least 1 negative result), but less likely to be documented and rarely altered antimicrobial therapy. “Sepsis unclear source” was the most likely indication to return only negative microbiology (22/35, 63%) (Table 1). Of 40 randomly sampled PEs with negative microbiology relevant to the prescription, 9 (23%) had the result documented in the medical records (compared with 27/33 (82%) for PEs with relevant positive microbiology; *P* = 6.7 × 10^− 6^, exact binomial test). In only 1 case did a negative result unambiguously lead to antimicrobial de-escalation (stopping ceftriaxone for meningitis following negative CSF results).

## Discussion

We have evaluated the patient-level impact of diagnostic microbiology on “review and revise” decision making in an unselected cohort of antimicrobial prescriptions for adult inpatients. Irrespective of indication, a substantial proportion (~ 40%) of patients started on systemic antimicrobials did not have diagnostic samples sent. This clearly limits the potential role of diagnostic microbiology in guiding antimicrobial review decisions. Where samples were sent, only ~ 20% yielded a significant positive result. Cultures may be negative for many reasons including prior antibiotic exposure in the patient, poor sampling technique, inadequate sampling equipment, delays in reaching the laboratory and insufficient test sensitivity for pathogen detection. While positive blood and CSF cultures usually impacted patient management, this often manifested as decisions to extend, broaden or intensify antimicrobial therapy (such as adding vancomycin after identifying Gram positive cocci in blood cultures). In such cases, the microbiological result may have been crucial for optimising patient therapy, but did not contribute to antimicrobial de-escalation. Positive sputum and urine cultures often did not significantly inform patient management and generally did not contribute to antimicrobial de-escalation. Among 40 reviewed PEs with negative microbiology we found only one instance in which the negative result directly informed a de-escalation decision.

There is a lack of direct evidence that microbiological test results support clinical decision making to limit antibiotic exposure. Our data are consistent with studies showing that cultures are often not taken from patients with suspected infections [8], but demonstrate further that when cultures are taken they still often fail to inform clinical decision making. The reasons why may depend on the clinical scenario. Given a standard treatment duration for lower UTI in women of 3 days, it is likely that time is a key limitation: results are generally available too late. Preliminary negative blood and sputum culture results are routinely available at 48 h and so the explanation here may be a lack of confidence in negative results by treating clinicians. Ensuring adequate equipment, technique and protocols are in place for collecting microbiological samples (e.g. taking uncontaminated blood cultures prior to administering antibiotics, clean midstream urine capture *etc*) may improve their diagnostic yield. However, even in the context of randomised controlled trials only 30–40% of patients with sepsis are bacteraemic [9]. Prescriber behavioural factors such as “prescribing etiquette” may also contribute to a reluctance to stop antimicrobials [10].

Our study has several limitations. The data were collected from a single centre; however, ours is a fairly typical NHS acute hospital with no reason to believe patients were selected in a biased way. We have focused on a specific, poorly studied, part of the pathway between diagnostic sampling and clinical decision making i.e. the actions based on the result taken by treating clinicians across a broad range of inpatient ward settings. We have not assessed whether the right patients are being sampled beyond the striking observation that 41% of patients started on systemic antibiotics weren’t sampled. We have not assessed quality of sampling e.g. blood culture taking and handling. Date information was sometimes lacking, but by allowing a +/− 7 day window we have if anything erred on the side of overestimating use of diagnostic testing. We have not assessed the impact of other microbiological sample types such as skin and wound swabs, stool, respiratory PCR or urinary pneumococcal antigen; but these comprise a minority of samples and are unlikely to make a substantial impact. Our findings are at odds with previous studies reporting benefit in antimicrobial selection through rapid microbiological identification with techniques such as MALDI-TOF [11]. However, such studies often fail to measure antibiotic de-escalation specifically, with limited evidence on the interpretation of negative results by treating clinicians across hospitals. In addition, antimicrobial therapy may initially get escalated following positive Gram stains (e.g. to cover potentially resistant organisms), then de-escalated (narrowed spectrum) once full antimicrobial susceptibility testing results become available, particularly for Gram negative pathogens [12], making it harder to summarise the net effect on antimicrobial exposure. We do not take account of antimicrobials that were never started due to negative microbiology results, which may be relevant in UTIs. Lastly we do not address the separate important role of diagnostic microbiology around screening for carriage of specific pathogens such as Carbapenem resistant Enterobacteriaceae (CRE) since our study focused on treatment rather than infection control practice. Of note none of these issues undermines our fundamental observation that in the majority of instances negative microbiology does not lead to clinicians stopping a patient’s antibiotic treatment.

## Conclusion

In conclusion, the impact of diagnostic microbiology in guiding antimicrobial de-escalation and reducing antimicrobial exposure on an individual patient level across hospital wards is currently limited. Our data suggest that an important reason is the perceived lack of predictive value for negative results by treating clinicians. For positive results, faster availability of antimicrobial susceptibility testing may also be impactful to limit unnecessary exposure to escalated empirical therapy. Negative microbiology is only part of the multifactorial assessment required for making antimicrobial de-escalation decisions, alongside patient factors, biochemistry and treatment response. Increasing the contribution of diagnostic microbiology to antimicrobial stewardship requires improvement in the actual and perceived negative predictive value of diagnostic testing.

## Supplementary information

**Additional file 1.** Supplementary Tables.

**Additional file 2.** Questionnaire used for reviews of Prescription Episodes.

## Data Availability

Data sharing is not applicable to this article which describes analysis of individual patient data as part of a service evaluation. No datasets were generated during the current study and patients did not consent to their individual data being shared.

## Abbreviations

BSUH: Brighton and Sussex University Hospitals NHS Trust
CRE: Carbapenem resistant Enterobacteriaceae
CSF: Cerebrospinal Fluid
ID: Identification
MALDI-TOF: Matrix-Assisted Laser Desorption/Ionization - Time-Of-Flight mass spectrometry
MRSA: Methicillin Resistant *Staphylococcus aureus*
NHS: National Health Service
PCR: Polymerase Chain Reaction
PE: Prescription Episode
UK: United Kingdom
USA: United States of America
UTI: Urinary Tract Infection
