# Supplementary material for: The impact of diagnostic microbiology on de-escalation of antimicrobial therapy in hospitalised adults
Source: BMC Infect Dis. 2020 Feb 3;20:102. doi: 10.1186/s12879-020-4823-4 (PMC6998081; doi:10.1186/s12879-020-4823-4)

The impact of diagnostic microbiology on de-escalation of antimicrobial therapy in hospitalised adults
William L. Hamilton, Sacha-Marie Pires, Samantha Lippett, Vikesh Gudka, Elizabeth Cross, Martin Llewelyn

Supplementary notes

The questionnaire used for all reviews of Prescription Episodes with positive pathogenic microbiology is shown below:

**Patient hospital ID:**

**Study ID:**

**Antibiotic prescription(s):**

**Indication:**

**Microbiology sample:**

**Microbiology result:**

*From medical notes/ prescription chart/ discharge summary:*

**1) Was the microbiology result clinically relevant to this prescription (Y/N):**

If answer to (1) was Yes, continue:

**2) Evidence of microbiology result acknowledged in medical notes (Y/N):**

**3) Evidence of direct microbiology involvement in case? (select one):**

**a) No evidence of microbiology involvement**

**b) Telephone only**

**c) Patient review from Microbiology doctor +/- telephone**

**4) Explicit evidence of microbiology result changing antimicrobial prescription (Y/N):**

**5) If (4) was Yes, how did the microbiology result change the prescription e.g. route, dose, duration, or antimicrobial agent. If there was a new agent, what was the change?**

The questionnaire used for all reviews of Prescription Episodes with negative microbiology is shown below:

**Patient hospital ID:**

**Study ID:**

**Antibiotic prescription:**

**Indication:**

**Microbiology sample:**

**Microbiology result:** Negative

*From medical notes/ prescription chart/ discharge summary. Circle yes or no:*

**1) Was the negative microbiology result relevant to this prescription: YES NO**

If answer to (1) was Yes, continue:

**2) Evidence of microbiology result acknowledged in medical notes: YES NO**

**3) On the day or next day that the negative result appeared on [Trust computer system], what happened with the antibiotic prescription?**

**NO CHANGE CHANGE AGENT CHANGE DOSE CHANGE ROUTE STOP**

**4) Did the negative result influence the decision made in q3?**

**YES PROBABLY POSSIBLY NO**

**5) Any other clinical impact that the negative result had?**

The standardised method for assessing whether negative microbiology impacted antimicrobial prescribing is shown below. ICE is the hospital’s computer reporting system for various diagnostic test results including microbiology.


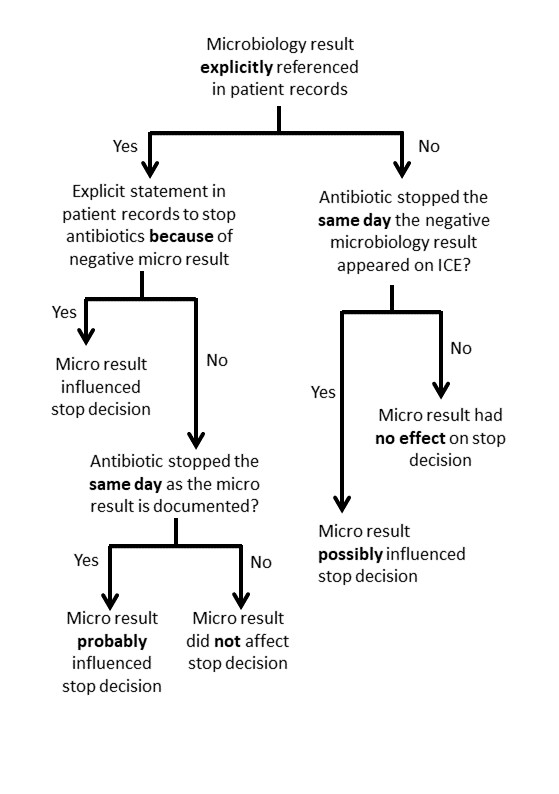

Supplement: Supplementary file 1 — Additional file 1. Supplementary Tables. [file 12879_2020_4823_MOESM1_ESM.docx]
